# Extra-axial inflammatory signal and its relationship to peripheral and central immunity in depression

**DOI:** 10.1093/brain/awae343

**Published:** 2024-12-10

**Authors:** Brandi Eiff, Edward T Bullmore, Menna R Clatworthy, Tim D Fryer, Carmine M Pariante, Valeria Mondelli, Lucia Maccioni, Nouchine Hadjikhani, Marco L Loggia, Michael A Moskowitz, Emiliano Bruner, Mattia Veronese, Federico E Turkheimer, Julia J Schubert

**Affiliations:** Department of Neuroimaging, Institute of Psychiatry, Psychology & Neuroscience, King’s College London, London SE5 8AF, UK; Department of Psychiatry, School of Clinical Medicine, University of Cambridge, Cambridge CB2 0SZ, UK; Cambridgeshire and Peterborough NHS Foundation Trust, Cambridge CB21 5EF, UK; Molecular Immunity Unit, Department of Medicine, University of Cambridge, Cambridge CB2 0AW, UK; Cambridge University Hospitals NHS Foundation Trust, and NIHR Cambridge Biomedical Research Centre, Cambridge CB2 0QQ, UK; Department of Clinical Neurosciences, School of Clinical Medicine, University of Cambridge, Cambridge CB2 0QQ, UK; Wolfson Brain Imaging Centre, University of Cambridge, Cambridge CB2 0QQ, UK; Department of Psychological Medicine, Institute of Psychiatry, Psychology & Neuroscience, King’s College London, London SE5 8AF, UK; Department of Psychological Medicine, Institute of Psychiatry, Psychology & Neuroscience, King’s College London, London SE5 8AF, UK; Department of Information Engineering, University of Padova, 35131 Padova, Italy; Department of Radiology, Athinoula A. Martinos Center for Biomedical Imaging, Massachusetts General Hospital, Harvard Medical School, Boston, MA 02129, USA; Department of Radiology, Athinoula A. Martinos Center for Biomedical Imaging, Massachusetts General Hospital, Harvard Medical School, Boston, MA 02129, USA; Department of Anesthesia, Critical Care and Pain Medicine, Massachusetts General Hospital, Harvard Medical School, Boston, MA 02114, USA; Department of Neurology, Massachusetts General Hospital and Harvard Medical School, Boston, MA 02114, USA; Department of Paleobiology, Museo Nacional de Ciencias Naturales (CSIC), 28006 Madrid, Spain; Alzheimer Center Reina Sofía, CIEN Foundation, ISCIII, 28031 Madrid, Spain; Department of Neuroimaging, Institute of Psychiatry, Psychology & Neuroscience, King’s College London, London SE5 8AF, UK; Department of Information Engineering, University of Padova, 35131 Padova, Italy; Department of Neuroimaging, Institute of Psychiatry, Psychology & Neuroscience, King’s College London, London SE5 8AF, UK; Department of Neuroimaging, Institute of Psychiatry, Psychology & Neuroscience, King’s College London, London SE5 8AF, UK

**Keywords:** inflammation, parameninges, skull, microglia, positron emission tomography, translocator protein

## Abstract

Although both central and peripheral inflammation have been observed consistently in depression, the relationship between the two remains obscure. Extra-axial immune cells may play a role in mediating the connection between central and peripheral immunity. This study investigates the potential roles of calvarial bone marrow and parameningeal spaces in mediating interactions between central and peripheral immunity in depression.

PET was used to measure regional TSPO expression in the skull and parameninges as a marker of inflammatory activity. This measure was correlated with brain TSPO expression and peripheral cytokine concentrations in a cohort enriched for heightened peripheral and central immunity comprising 51 individuals with depression and 25 healthy controls.

The findings reveal a complex relationship between regional skull TSPO expression and both peripheral and central immunity. Facial and parietal skull bone TSPO expression showed significant associations with both peripheral and central immunity. TSPO expression in the confluence of sinuses was also linked to both central and peripheral immune markers. Group-dependent elevations in TSPO expression within the occipital skull bone marrow were also found to be significantly associated with central inflammation.

Significant associations between immune activity within the skull, parameninges, parenchyma and periphery highlight the role of the skull bone marrow and venous sinuses as pivotal sites for peripheral and central immune interactions.

## Teaser

Individuals with depression often show signs of inflammation in both the brain and body. Eiff *et al.* provide evidence that the skull bone marrow is involved in the crosstalk between the central and peripheral immune systems, with this crosstalk likely contributing to the inflammation observed in depression and other diseases.

**See Wimberley and Thompson (https://doi.org/10.1093/brain/awae403) for a scientific commentary on this article.**

## Introduction

Ample evidence supports the theory that major depressive disorder is, at least in part, linked to immune dysfunction.^1^ Heightened peripheral and central inflammation have been observed consistently among major depressive disorder cohorts.^1-12^ Although significant elevations in peripheral and central inflammatory markers have been observed, they often do not appear to be directly correlated,^7-10,13^ and the link between the two phenomena remains unclear. Therefore, this work investigates whether an intermediate component is involved in the interplay between systemic and central inflammation in depression and whether immune cells acting directly on the brain are sourced more locally from extra-axial regions.

Supporting the theory of an inflammatory aetiology of depression, numerous meta-analytical studies report elevated levels of peripheral pro-inflammatory cytokines in patients with depressive disorders.^2-6^ Although previous evidence shows a dysregulation of nearly all immune markers within these cohorts,^2^ C-reactive protein (CRP), interleukin (IL)-6 and tumour necrosis factor (TNF)α are among the most reliable inflammatory biomarkers adopted in depression studies.^1^ The production pathways of these acute-phase proteins are all interconnected, with increased concentrations of some regulating the release of others, ultimately resulting in systemic inflammation. TNFα is a major pro-inflammatory cytokine produced by dendritic cells and macrophages and is involved in activating downstream inflammatory cascades, including the release of IL-6 from macrophages.^2,3^ IL-6, which can have pleiotropic functions pertaining to immune modulation, is produced by a myriad of cell types within the periphery and the brain.^14^ Following this specific lineage, the release of IL-6 from macrophages triggers the production of CRP by liver hepatocytes.^15^ Although clinical and immunopsychiatric research primarily uses CRP as a marker of inflammation, other markers might better capture the systemic immune dysfunction observed in depression.^16^

The relationship between depression and peripheral inflammation appears to have a bidirectional nature.^17^ The development of depressive symptoms is a prevalent side effect of immunotherapy, as observed when interferon-α is administered to individuals being treated for non-psychiatric illnesses.^18,19^ Conversely, anti-inflammatory medications have demonstrated efficacy in reducing depressive symptoms and improving antidepressant treatment response in some cases,^20^ particularly among patients with elevated peripheral CRP values.^21,22^

Substantial evidence also points to a notable increase in central inflammation among individuals with major depressive disorder.^1,7-12^ Microglia, the primary regulators of brain immune activity in both physiological and pathological conditions, play a pivotal role.^23^ The 18 kDa outer mitochondrial translocator protein (TSPO) displays consistent upregulation in various cell types conducive to neuroinflammatory activity, including microglia, activated astrocytes, mast cells, macrophages and leucocytes.^24-29^ PET studies using radiotracers specific to TSPO have revealed evidence of neuroimmune activation across numerous neurological and psychiatric disorders.^30^ Notably, in major depressive disorder, increased TSPO expression has been reported within brain regions involved in mood regulation, including the anterior cingulate cortex (ACC), prefrontal cortex (PFC) and insula.^7,9^

Many researchers have investigated whether increased inflammatory activity in the brain is linked to peripheral immune responses.^31,32^ However, as reviewed by Turkheimer *et al*.,^13^ much of the available data from human cohorts does not show a direct correlation between peripheral immune activation, as measured by plasma cytokines, and neuroinflammation, as measured by TSPO-PET. This observation has prompted a shift in focus towards understanding the functional role of extra-axial immune cells, originating from both the skull bone marrow and the meninges, in mediating the connection between systemic and central inflammation.

Such interest stems from compelling evidence from several recent preclinical studies that implicates immune cell trafficking across the skull and meninges in neuroinflammatory responses.^33-39^ Localized cell tagging of leucocytes has revealed that neutrophils recruited to inflamed cerebral tissue following stroke were derived primarily from skull bone marrow rather than distal tibial marrow.^35^ Additionally, neutrophils were shown to migrate from the skull to the brain, against typical blood flow, via direct vascular channels connecting skull bone marrow to the dura. Further investigation into this system has revealed that myeloid cell reservoirs exist within skull bone marrow which, in homeostatic conditions, supply monocytes and neutrophils to the dural meninges.^34^ Moreover, during pathological states, meningeal niches dispense immune cells into brain parenchyma and are replenished by adjacent skull bone marrow, all seemingly independent of blood circulation or other peripheral immune responses. Importantly, communication along the skull–meninges–brain axis appears to be bidirectional, meaning that inflammatory signalling molecules from the brain carried through CSF also enter the skull, resulting in exacerbated immune responses from the skull bone marrow.^35,36,39^ Together, this evidence suggests that both the skull bone marrow and meninges play crucial roles in brain immunity, encompassing both homeostatic maintenance and responses to pathological conditions.

Pro-inflammatory cells entering the brain appear to be sourced directly from adjacent regions in the skull and meninges rather than the periphery. This hypothesis has been confirmed in a preclinical model of depression^37^ and could explain why previous studies have been unable to find significant direct correlations between central and peripheral inflammation. Hence, the link between skull, meningeal and parenchymal immune compartments may provide a new perspective on inflammatory responses in major depressive disorder in addition to new therapeutic targets.

Extra-axial inflammatory responses during human neurological disease have recently been investigated using TSPO-PET. Disease-specific patterns of TSPO signals have been detected in the skull within stroke, multiple sclerosis and neurodegenerative disease patients.^36^ In a study performed by Hadjikhani *et al*.^40^ investigating the inflammatory correlates of migraine with visual aura, TSPO-PET signal was elevated within the meninges and skull bone marrow (parameningeal tissue) overlying the occipital lobe. These findings, along with evidence of vasculature linking the skull and parenchyma, suggest that an important interplay exists between upregulated inflammatory responses within the skull and the pathophysiology of diseases associated with neuroinflammation.^40^ This also may relate to previous findings of parameningeal immune-mediating cell migration into the brain in a preclinical model of depression.^37^

These studies have provided a valuable framework for the present study to investigate whether these disease-associated patterns of TSPO expression in the calvarial bone marrow are echoed in a neuropsychiatric condition known to have an inflammatory component, such as depression. Hence, in this work, we investigated the association between the inflammatory status of parameningeal spaces and peripheral and central immunity in a large cohort of depressed subjects enriched for peripheral cytokines and neuroinflammatory activity. Correlations were then computed to examine the relationship between TSPO expression in the skull and parameningeal regions and both brain TSPO expression and peripheral cytokine concentrations.

## Materials and methods

### Participants

The study cohort comprised 51 depressed individuals (DP) aged 25–50 years and 25 age-matched healthy controls (HC). Recruitment for this investigation was conducted across an array of clinical research sites in the UK, as part of the BIODEP (Biomarkers in Depression) study under the NIMA consortium (https://www.neuroimmunology.org.uk/biodep/). Depressed individuals recruited for this study exhibited a total Hamilton Depression Rating Scale (HDRS) score of ≥13. Depressed subjects representing a range of peripheral inflammatory states, as indicated by CRP levels, were enrolled. CRP concentration was adopted as a measure of the severity for the peripheral inflammatory state, and depressive patients were categorized into high- and low-CRP groups using a predefined CRP concentration threshold of 3 mg/l. The HC participants were matched in terms of mean age to the DP and had no personal history of treated clinical depression.

Exclusion criteria for both the DP and HC participants included no documented history of neurological disorders, absence of current drug and/or alcohol abuse, no recent participation in clinical drug trials, absence of concurrent medication or medical conditions that could introduce confounding variables into result interpretation, and no current status of pregnancy or breastfeeding. Ethical endorsement for this study was secured from the National Research Ethics Service Committee East of England—Cambridge Central (REC reference: 15/EE/0092), and the research design adhered to the directives set forth by the UK Administration of Radioactive Substances Advisory Committee. Before data collection commenced, all participants granted written informed consent. Demographic and clinical characteristics are presented in detail in Table 1. Further insights into the dataset leveraged for this study can be referenced from the primary publication of the ^11^C-PK11195 PET results concerning this specific cohort.^9^

**Table 1:** Demographic, clinical and scanning characteristics

Variablemean (SD)	Depressed(*n* = 51)	Healthy(*n* = 25)	*P*-value
**Demographic**			
Age, years	36.2 (7.3)	37.3 (7.8)	0.561
Male, *n* (%)	15 (29)	11 (44)	0.208
Weight, kg	80.3 (14.4)	73.7 (15.1)	0.072
BMI, kg/m^2^	27.7 (4.0)	24.8 (3.9)^a^	0.001
**Clinical**			
CRP, mg/l	2.9 (2.8)	1.1 (0.9)	< 0.001
HDRS	18.5 (3.7)	0.6 (0.9)	<0.001
State anxiety score	50.7 (9.8)	25.7 (6.2)	<0.001
Trait anxiety score	60.9 (7.9)	28.9 (5.8)	<0.001
**Scanning**			
Dose, MBq	360.3 (53.2)	376.2 (44.8)	0.138
Injected mass, mg	3.0 (1.6)	3.4 (1.8)	0.486
Specific activity, GBq/mmol	50.80 (21.33)	50.56 (25.94)	0.711
Total motion, mm	7.76 (3.75)	7.27 (3.39)	0.562
Max interframe motion, mm	1.62 (0.90)	1.45 (0.68)	0.532
Scan start time	15:14:44	14:58:05	0.761

BMI = body mass index; CRP = C-reactive protein; HDRS = Hamilton Depression Rating Score.

^a^BMI was not available for one control subject due to missing height measurement.

### Clinical assessments

Venous blood samples were collected from the antecubital region to measure levels of circulating peripheral inflammatory proteins and pro-inflammatory cytokines, including CRP, TNFα and IL-6. Blood collection took place in the morning between 08:00 and 10:00 h on the day of clinical assessment. To ensure standardized conditions, participants were instructed to fast for 8 h and refrain from physical exercise for 72 h before the blood sampling session. Subjects were required to lie in a supine position for 30 min before venous blood sample collection.

CRP levels were determined using a turbidimetry method conducted on Beckman Coulter AU analysers, using latex particles coated with anti-CRP antibodies.^41^ For immune-related protein measurements, plasma preparation tubes (BD Cat. No. 362799) were centrifuged at 1600*g* for 15 min at room temperature, and the resulting plasma supernatant was promptly frozen at −80°C. Upon thawing, markers were assayed in duplicate using the Pro-Inflammatory Panel 1 (K15049D) and Cytokine Panel 1 (K150150D) V-PLEX 10-spot immunoassay kits from Meso Scale Discovery, according to the manufacturer’s instructions (MSD). Only samples with immune plasma concentrations equal to or exceeding the lower limit of detectability were included, which was 0.06 pg/ml for IL-6 and 0.05 pg/ml for TNFα. The assay coefficients of variability were consistently <15% for both biomarkers.^16^ Three healthy controls and one depressed subject were excluded owing to unavailable IL-6 and TNFα measurements.

### PET and MRI data acquisition

All participants underwent an imaging protocol consisting of a 60 min simultaneous dynamic PET scan and high-resolution T_1_-weighted brain MRI scan following the administration of ^11^C-PK11195 (mean injected dose, 361 ± 53 MBq) using a GE SIGNA PET/MR scanner (GE Healthcare). For attenuation correction, a multi-subject atlas method was used, incorporating enhancements for the MRI brain coil component.^42-44^ Concurrently, corrections for scatter, randoms, normalization, sensitivity, dead time and decay were applied directly on the scanner. The dynamic sinograms were subsequently reconstructed into arrays with dimensions of 128 × 128 × 89, yielding a voxel size of 2 mm × 2 mm × 2 mm, using the time-of-flight ordered subsets expectation maximization method, with parameters set at six iterations, 16 subsets and no smoothing.

### Image processing and quantification

Image preprocessing was performed using MIAKAT software (version 4.2.6; http://www.miakat.org/MIAKAT2/index.html) within MATLAB (version R2015b; The MathWorks, Inc., Natick, MA, USA) and was completed as part of the original PET processing for the BIODEP study.^9^ Briefly, time activity curves were extracted from the bilateral ACC region using the CIC v.2.0 neuroanatomical atlas transformed to subject image space. The ACC was chosen as the regional marker for brain inflammation based on previous results from this dataset.^9^ A more detailed account of image collection and initial processing can be found in the original report of the BIODEP PET data.^9^

**Figure 1:**
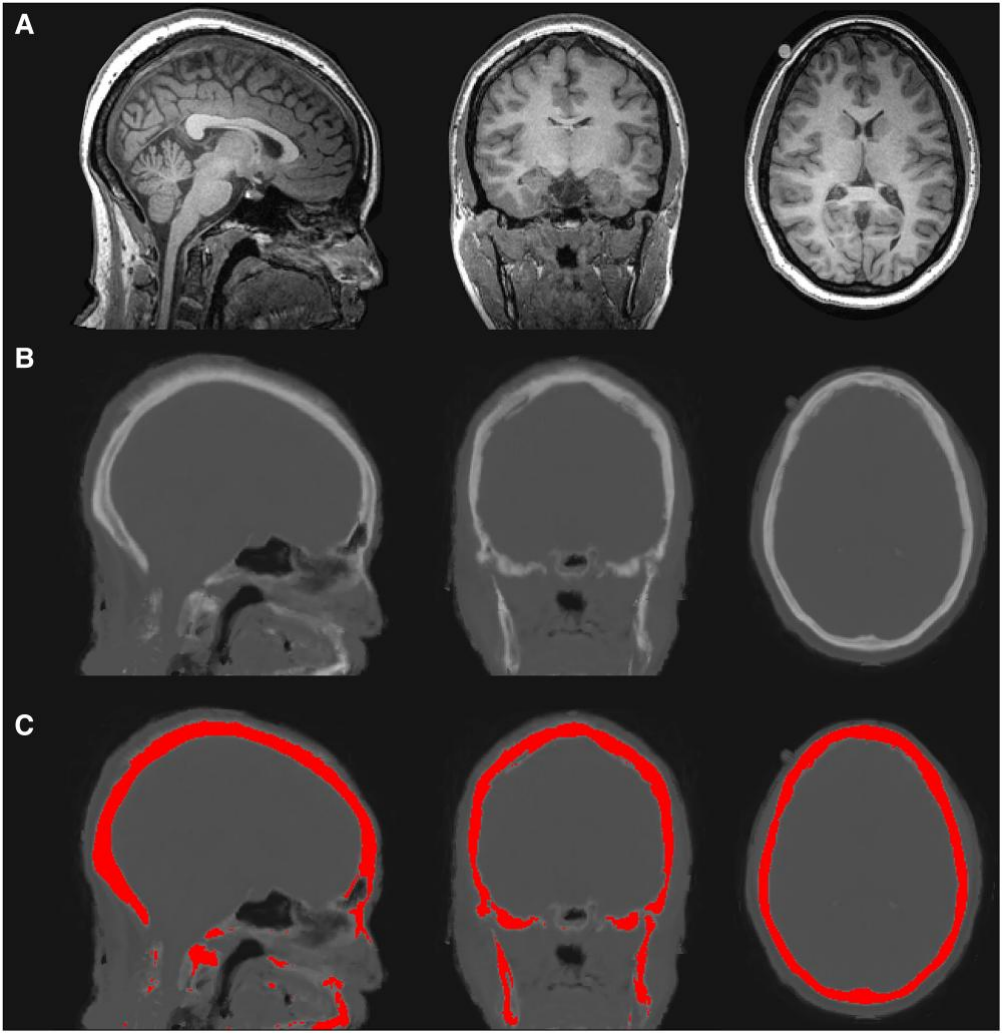
**Generation of skull mask from pseudo-CT images.** (**A**) T_1_ structural brain image. (**B**) Psuedo-CT (pCT) image synthesized from T_1_ structural brain image. (**C**) pCT image with binary skull mask overlaid. Skull voxels were identified by applying a lower intensity threshold (600 Hounsfield units). Gaps and voids in the binarized image were filled to create a complete skull mask.

Pseudo-CT (pCT) images were synthesized from each subject’s T_1_ structural brain image using a fully automated online software package (http://niftyweb.cs.ucl.ac.uk/).^42,43^ To isolate voxels primarily representing the skull, a predefined lower intensity threshold of 600 Hounsfield units was applied to the pCTs. The threshold was chosen heuristically based on voxel values in the skull and surrounding areas. Gaps or voids in the binarized image were filled to create a complete skull mask (Fig. 1).

A parameningeal tissue template devised as part of the previously discussed migraine study was provided by the HST/MGH A. A. Martinos Center for Biomedical Imaging.^40^ The regions of interest (ROIs) included in this template consisted of the skull bone marrow and dura mater overlying the occipital lobe, parietal lobe, orbitofrontal cortex and dorsolateral prefrontal cortex (DLPFC).

The parameningeal tissue ROI templates were provided in 2 mm isotropic MNI space, and all ROIs originally had the same volume (100 voxels, 800 mm^3^). Additional processing of all ROIs was performed primarily using MATLAB version R2018b and associated toolboxes. Using SPM12 software, the templates were resliced to 1 mm isotropic resolution and non-linearly warped to align with the structural MRI of individual subjects. These warped parameningeal tissue templates were then dilated using a spherical kernel with radius of 3 voxels and masked using the previously defined skull ROI. This masking step was carried out to enhance the accuracy of the ROIs in depicting the regions of the skull. The ROIs were then eroded using a spherical kernel with radius of 1 voxel to mitigate the effects of partial volume of surrounding tissues. The warped and masked parameningeal tissue templates were then re-sliced again to 2 mm isotropic resolution to align with the subjects’ dynamic PET. It should be noted that the ROI signal analysed in this study is associated with gross regions of the bone covering the DLPFC, parietal and occipital cortex. These bone regions have no established homological value or position and must therefore be intended as a general sample of the frontal, parietal and occipital squamae, respectively. In this sense, we use the terms ‘DLPFC skull’, ‘parietal skull’ and ‘occipital skull’ not to refer to the whole bones, but only to a minor central part of the overlying bones sampled in this study.

Segmentation was performed using FreeSurfer v.6.0 software, using as input the T_1_ structural brain images that had previously been co-registered to the subjects’ PET native space. A binary mask delineating non-brain areas was derived from FreeSurfer output by subtracting a binarized mask, obtained from the FreeSurfer parcellation image (aparc.a2009s + aseg_bin.nii.gz), from the FreeSurfer brainmask image.

**Figure 2:**
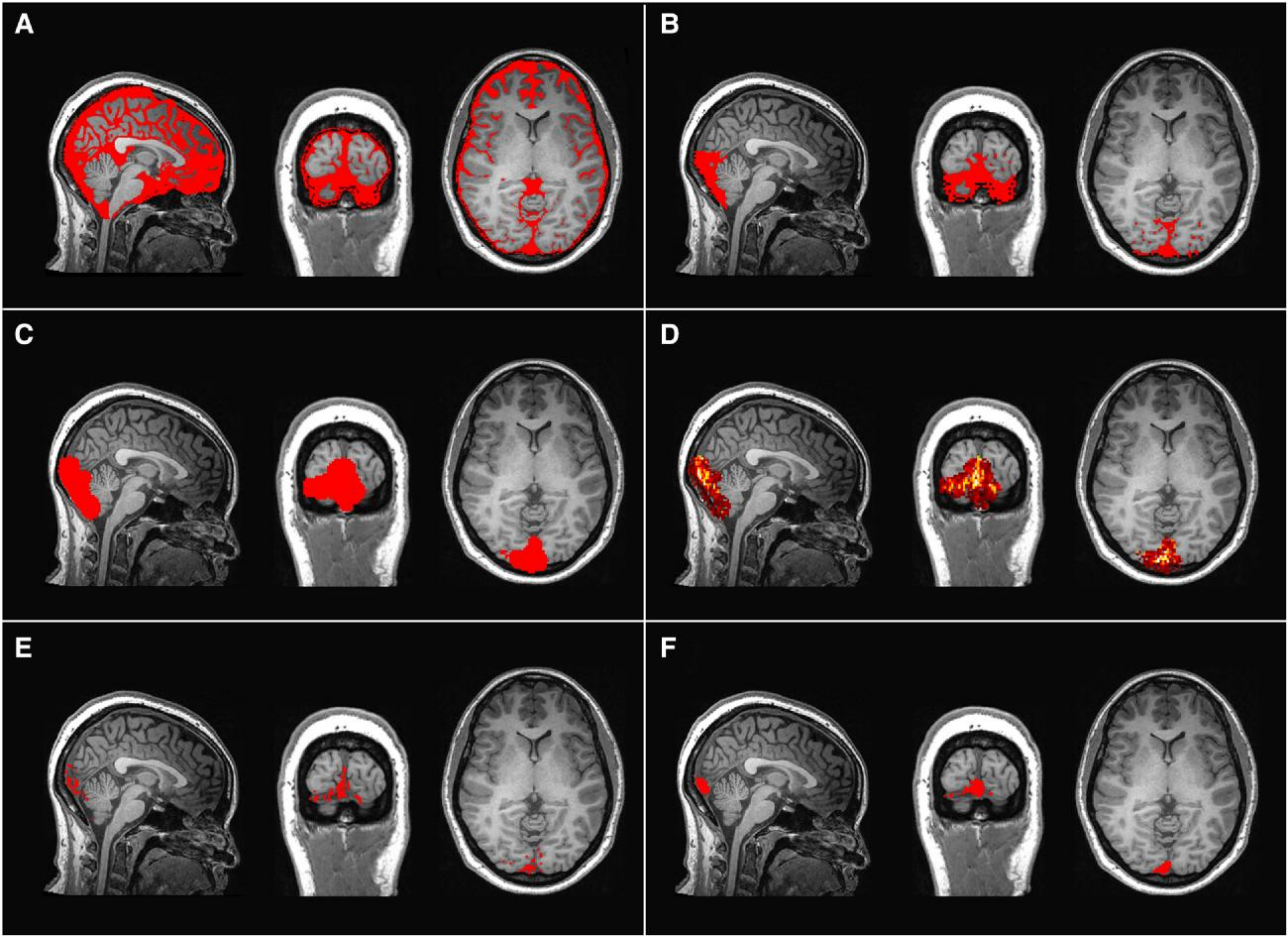
**Confluence of sinuses region of interest generation process.** (**A**) Binary mask delineating non-brain areas, overlaid on T_1_ structural brain image. (**B**) Refinement of the mask to isolate the posterior cranial fossa. (**C**) Exclusion of small clusters and dilatation of retained clusters to ensure coverage of the confluence of sinuses. (**D**) Using PET data to identify voxels exhibiting ≥25% of the peak activity observed in the mode peak frame time, indicating a predominant presence of blood in those voxels. (**E**) Only voxels identified as predominantly containing blood are retained in the binary mask. (**F**) Removal of smaller noise clusters, refinement of mask edges through dilatation and erosion, and retention of the largest volume cluster to produce the final binary mask representing the confluence of sinuses.

This mask, initially encompassing all non-brain spaces, was further refined to isolate the posterior cranial fossa specifically. This region houses the confluence of sinuses, which is recognized as the largest venous blood pool, enabling the quantification of TSPO expression while mitigating the effects of partial volume. Clusters smaller than 5 voxels within the posterior non-brain mask were excluded, and the retained clusters were further dilated with a spherical kernel having a radius of 4 voxels to ensure full coverage of the confluence of sinuses. Voxels were considered to belong to the same cluster if they shared at least one edge. The posterior cranial fossa mask was applied to the dynamic PET data to focus the analysis on this specific region. The individual PET frame containing the highest number of voxels at their peak activity within the masked area was identified as the mode peak frame time. An intensity threshold was applied to this masked frame, retaining only those voxels with activity levels ≥25% of the maximum observed activity. The kinetics of these voxels strongly suggest that they predominantly contain blood. Smaller clusters containing <5 voxels, attributed to noise, were removed from the mask. To refine the edges of the mask, a process was applied involving dilatation followed by erosion using a spherical kernel with a radius of 2 voxels. Finally, only the largest volume cluster was retained in the mask, resulting in the final binary mask representing the confluence of sinuses ROI (Fig. 2). This method for segmenting the confluence of sinuses has been adapted from the original technique used for segmenting the carotid siphons for extracting the image-derived input function.^45^

**Figure 3:**
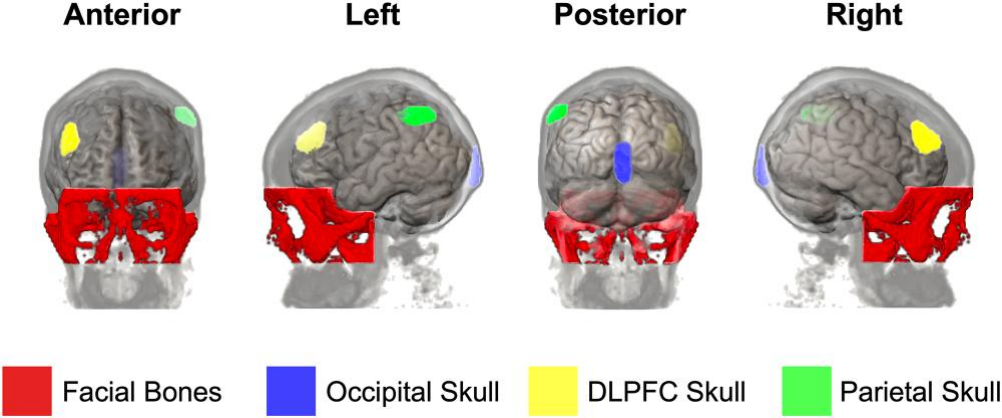
**Three-dimensional representation of skull regions of interest.** See also Supplementary Fig. 1 for the three-dimensional rendering depicting specific regions of interest within the skull. Regions of interest were informed by the template provided by Hadjikhani *et al*.^40^ and pseudo-CT renderings derived from participants’ structural MRIs. The use of pseudo-CTs facilitated the refinement of these regions, ensuring that voxels represented bone marrow exclusively, without including adjacent dura mater. DLPFC = dorsolateral prefrontal cortex.

A facial bone ROI was defined by excluding voxels associated with the neurocranium while specifically including bones belonging to the upper viscerocranium. This resulted in an ROI comprising the glabella, nasal bones, zygomatic bones, lacrimal bones, coronoid processes of the mandible, ethmoid bone, sphenoid bone, vomer and the maxillary body. All ROIs (Fig. 3 and Supplementary Fig. 1) underwent thorough visual inspection for quality assurance, and manual adjustments were made as necessary to ensure the accurate representation of intended tissues within the ROIs.

Time activity curves were extracted for each subject across occipital, DLPFC and parietal skull regions, facial bone and the confluence of sinuses ROIs, in addition to the ACC. Quantification of ^11^C-PK11195 binding within these regions was accomplished using standardized uptake values (SUVs), using time activity curve data from 5 min to the end of the scan time in order to mitigate the impact of early frame noise and tracer delivery. Group average SUV curves for all ROIs are provided in Supplementary Fig. 2. Note that in previous studies intra-axial activity was normalized to a brain reference region; in this work this approach was avoided to ensure consistency in the quantification method for both extra- and intra-axial regions.

### Statistical analysis

All statistical analyses were carried out using SPSS software (version 28.0.1.1; IBM, Armonk, NY, USA). The normality of all dependent variables was assessed using the Shapiro–Wilk test. Group differences in trait anxiety and body weight were analysed using the independent-samples *t*-test. All other group differences in experimental variables, encompassing demographic, clinical and scanning characteristics, were evaluated using the independent-samples Mann–Whitney U-test (Table 1). An extreme outlier with a notably high TNFα value was identified in one DP case and excluded from further analysis.

A repeated-measures general linear model was used, treating extra-axial regional average SUVs (facial bones, occipital skull, DLPFC skull, parietal skull and confluence of sinuses) as the repeated measure, subject group as the fixed factor, and TNFα, IL-6, CRP and ACC SUV as covariates. Before being included in statistical analyses, CRP and cytokine values underwent log_10_ transformation to achieve an approximately normal distribution. Only variables that exhibited significant contributions (*P*-value ≤0.05) to the statistical model were retained in the final model.

Additionally, a univariate analysis of covariance (ANCOVA) was conducted for the average SUV in each region (facial bones, occipital skull, DLPFC skull, parietal skull and confluence of sinuses), with subject’s group as the fixed factor and TNFα and ACC SUV as covariates. Spearman’s correlation was conducted to investigate the relationship between TNFα and ACC SUV. All *P*-values are reported without correction for multiple comparisons.

## Results

A repeated-measures general linear model was performed to determine whether there were statistically significant group differences in TSPO signal across extra-axial regions and whether central and peripheral inflammatory markers contributed to these variations. Initially, this test was performed including CRP, IL-6, TNFα and ACC SUV as covariates, with the group as a between-subjects factor. However, tests of between-subjects effects revealed that CRP and IL-6 did not have a significant main effect on TSPO signal variability across regions [CRP: *F*(1,64) = 0.981, *P* = 0.326, *ηp*^2^ = 0.015; IL-6: *F*(1,64) = 1.572, *P* = 0.215, *ηp*^2^ = 0.024] and were therefore excluded from the final model.

The general linear model was performed again including TNFα and ACC SUV as covariates and group as a between-subjects factor. Mauchly’s test of sphericity indicated that the assumption of sphericity was violated [*χ*^2^(9) = 84.928, *P* < 0.001] and therefore Greenhouse–Geisser correction was applied (*ε* = 0.657). Results from the general linear model indicated that TNFα, ACC SUV and group contributed significantly to variation in extra-axial inflammation between subjects [TNFα: *F*(1,67) = 4.747, *P* = 0.033, *ηp*^2^ = 0.066; ACC SUV: *F*(1,67) = 14.263, *P* < 0.001, *ηp*^2^ = 0.176; group: *F*(1,67) = 4.005, *P* = 0.049, *ηp*^2^ = 0.056]. A significant interaction effect was also observed between the extra-axial region and ACC SUV [*F*(2.628,176.083) = 3.312, *P* = 0.027, *ηp*^2^ = 0.047], prompting additional univariate analyses within each specific extra-axial region.

**Figure 4:**
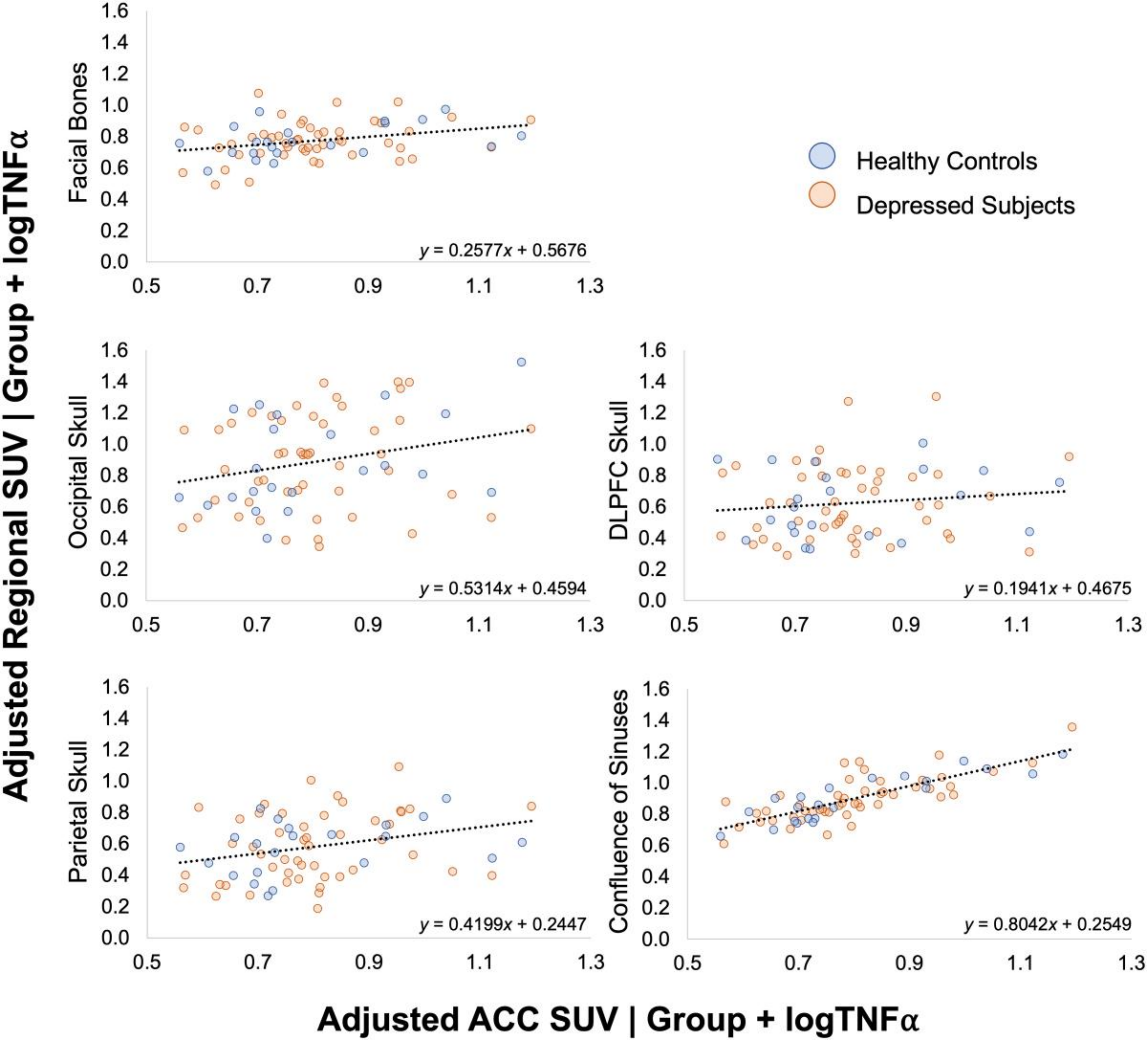
**ACC SUV effect on regional extra-axial SUV.** Partial regression plots presenting the relationship between ACC SUV and regional extra-axial SUV, accounting for group and logTNFα. ACC = anterior cingulate cortex; SUV = standardized uptake value.

Univariate ANCOVA was used to assess whether inflammatory signal within the individual extra-axial ROIs was significantly influenced by peripheral and central inflammation, represented by TNFα and ACC SUV, respectively, and group. ACC SUV had a significant positive effect on SUVs within the occipital skull [*F*(1,67) = 4.667, *P* = 0.034, *ηp*² = 0.065], facial bones [*F*(1,67) = 7.546, *P* = 0.008, *ηp*^2^ = 0.101], parietal skull [*F*(1,67) = 6.504, *P* = 0.013, *ηp*^2^ = 0.088] and the confluence of sinuses [*F*(1,67) = 118.883, *P* = <0.001, *ηp*^2^ = 0.640] (Fig. 4). These values indicate that TSPO signal within the ACC is significantly correlated with TSPO signal in these regions, irrespective of group differences, with the most robust association found in the confluence of sinus region (Table 2).

**Table 2:** Overview of univariate results

	Facial bones	Occipital	DLPFC	Parietal	Confluence
log_10_TNFα (*β* | *P*-value)^a^	0.261 | 0.033*	0.280 | 0.775	0.344 | 0.169	0.591 | 0.007*	0.342 | <0.001*
ACC SUV (*β* | *P*-value)^b^	0.258 | 0.008*	0.531 | 0.034*	0.194 | 0.320	0.420 | 0.013*	0.804 | <0.001*
Group (*P*-value)^c^	0.057	0.021*	0.169	0.117	0.992
*R* ^2^	0.202	0.131	0.054	0.213	0.661
Adjusted *R*^2^	0.166	0.092	0.012	0.177	0.646

ACC SUV = anterior cingulate cortex standardized uptake value; Confluence = confluence of sinuses; DLPFC = dorsolateral prefrontal cortex; TNFα = tumour necrosis factor α. Significant differences (*P* < 0.05) are indicated by an asterisk.

^a^Results are adjusted for effect of ACC SUV and diagnostic group.

^b^Results are adjusted for effect of log_10_TNFα and diagnostic group.

^c^Results are adjusted for effect of log_10_TNFα and ACC SUV.

**Figure 5:**
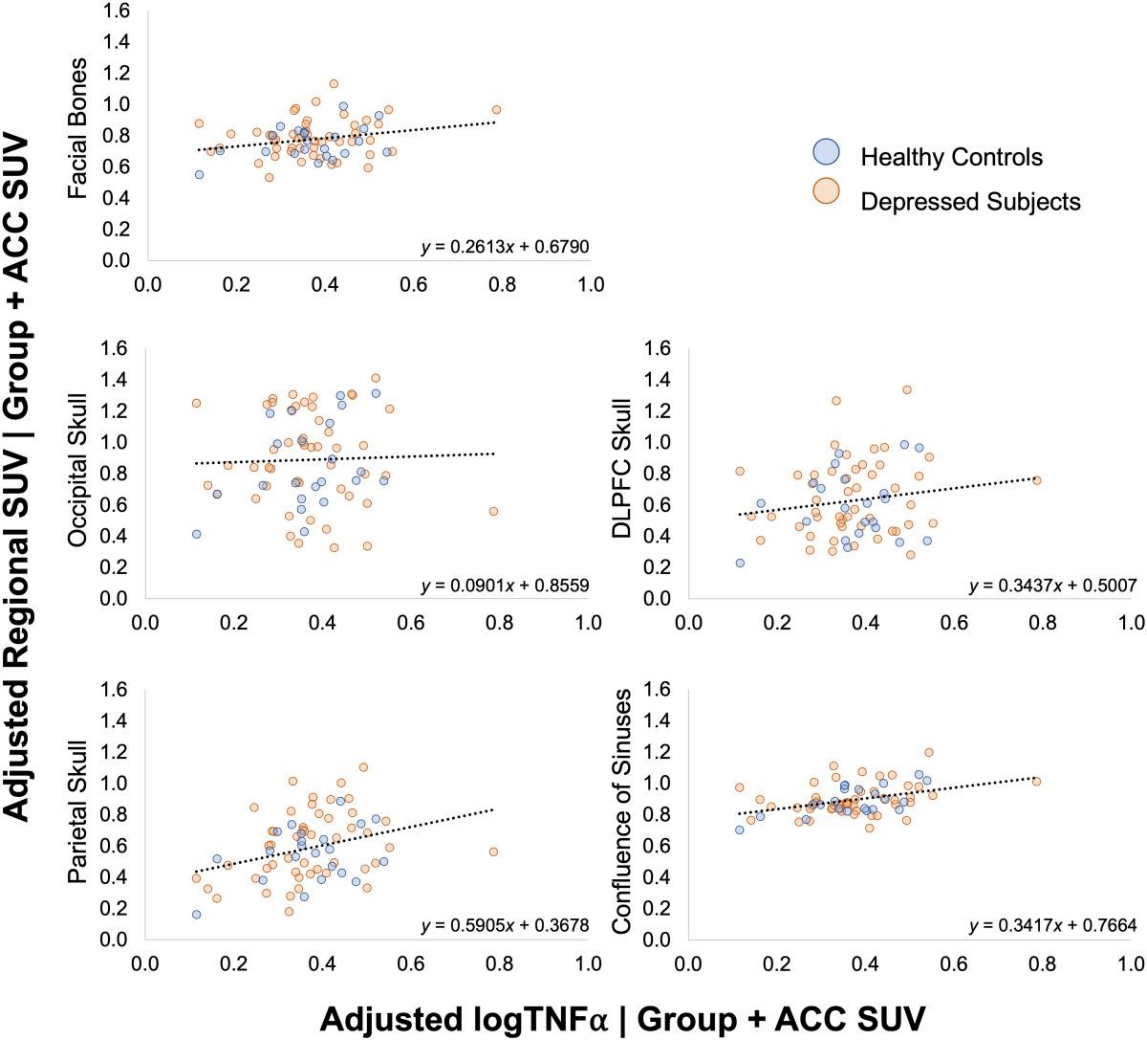
**LogTNFα effect on regional extra-axial SUV.** Partial regression plots presenting the relationship between logTNFα and regional extra-axial SUV, accounting for group and ACC SUV. ACC = anterior cingulate cortex; SUV = standardized uptake value.

TNFα also has a significant positive effect on SUV variance within the facial bones [*F*(1,67) = 4.751, *P* = 0.033, *ηp*^2^ = 0.066], parietal skull [*F*(1,67) = 7.876, *P* = 0.007, *ηp*^2^ = 0.105] and confluence of sinuses [*F*(1,67) = 13.142, *P* < 0.001, *ηp*^2^ = 0.164] (Fig. 5). No significant association was found between TNFα and ACC SUV [*r*(71) = −0.072, *P* = 0.553].

**Figure 6:**
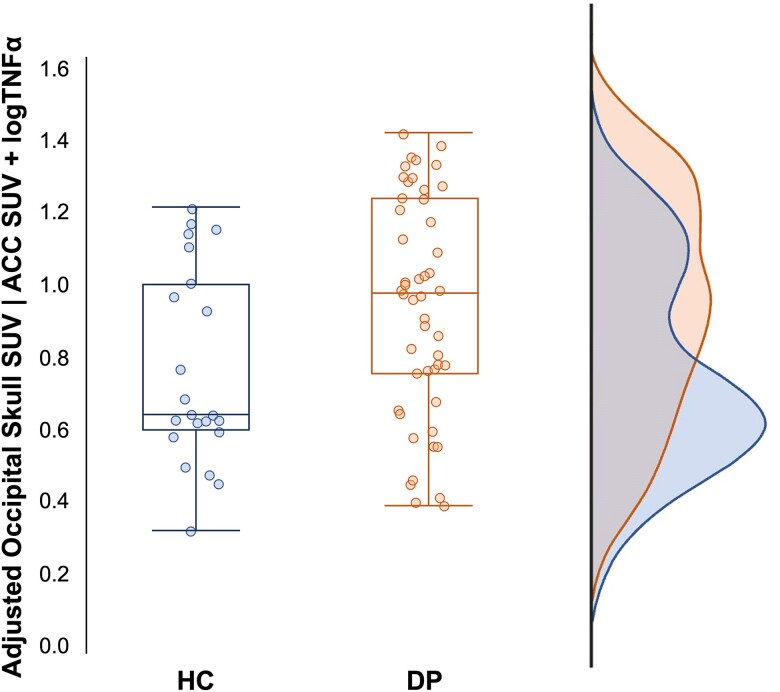
**Group effect on occipital skull SUV.** Box-and-whisker plot representing group differences in occipital skull SUV, adjusted for ACC SUV and logTNFα, with all data-points shown. The horizontal line within the boxes indicates the median, each box represents the values within the 25th–75th percentile, and whiskers show the range of SUVs. ACC = anterior cingulate cortex; DP = depressed individuals; HCv healthy controls; SUV = standardized uptake value.

The univariate analysis of covariance revealed a significant main effect of group on occipital skull SUV only [*F*(1,67) = 5.605, *P* = 0.021, *ηp*^2^ = 0.077]. Participants in the depression group exhibited higher occipital skull SUVs [mean = 0.975, standard error of the mean (SEM) = 0.043] than controls (mean = 0.791, SEM = 0.064), after adjusting for ACC SUV and TNFα (Fig. 6).

## Discussion

The present study examined the relationship between skull bone marrow immune activity and both peripheral and central inflammation within a cohort of depressed individuals and healthy controls. The dataset included a diverse range of peripheral and central inflammatory states, as evidenced by CRP levels and parenchymal TSPO expression, respectively, providing valuable insights into the interplay between these factors. The results suggest that calvarial marrow, the source of TSPO signal in the skull, and venous blood pool are associated with both peripheral and central immune signals. However, this association varies regionally.

A robust significant association was observed between the TSPO-PET signal within the confluence of sinuses and the parenchymal ACC inflammatory signal, and with peripheral TNFα concentrations. Located along the occipital bone, the confluence of sinuses plays a vital role in draining endocranial blood, generally through the transverse or occipito-marginal sinuses, often both converging into the jugular canals.^46-48^ Accordingly, whatever physiological product is introduced into this sinus is quickly drained out of the braincase. Thus, it is reasonable to infer that the signal detected may be associated with the perivascular space of the sinus, and not only with the sinus itself. The dural sinuses appear to be critically involved in immune cell surveillance, recruitment and trafficking, acting as an interface facilitating interactions between the central and peripheral immune systems.^49^ The dural lymphatics likely also contribute to this connection, as they have previously been observed to form an extensive network at the base of the skull proximal to the dural venous network.^50^ Notably, ACC SUV and TNFα measures, representing central and peripheral immune activity, respectively, did not exhibit a direct correlation with each other. This finding aligns with previous reports that have struggled to establish a direct link between central and peripheral immunity.^9,13,51^ However, both ACC SUV and TNFα showed strong positive associations with TSPO signal in the confluence of sinuses. It is also noteworthy that when analysing whether these same results are present within an arterial blood pool, such as the carotid siphons, this result was not observed. Rather, blood within the carotids was significantly correlated only with central immune signal and not with peripheral immune signal. Details about this additional analysis using the carotid siphons can be found in the Supplementary material, Methods and Results sections. Together, these observations underscore the venous blood pool as an important hub for central–peripheral immune crosstalk.

We observed weaker associations with peripheral and central immunity in the marrow of facial bones and the parietal skull. The recently uncovered network of microscopic vascular channels, documented in frontal, parietal and occipital skull regions, serves as a conduit for communication between these systems.^33,35,38^ Regional variations in channel length, width and density^39,52,53^ may account for our disparate findings across ROIs. Our finding in parietal skull and facial bones suggests a subtle intermediary communication between central and peripheral systems. Notably, this study marks the first exploration of the potential involvement of facial bones in this context. The investigation was prompted by the proximity of facial bones to the nasal passages and sinuses, housing the nasal microbiota crucial for immune regulation and general CNS homeostasis.^54,55^ The TSPO signal in facial bones may be sensitive to changes in these spaces; however, further research is needed to elucidate the connection between facial bones and nasal passages and its implications for the brain–periphery connection.

A significant positive association with parenchymal ACC TSPO was found in the occipital skull region. A large haematopoietic niche containing a variety of immune cells has been observed within the caudal region of the skull in mouse models,^38^ which, in humans, translates to the occipital region. Moreover, in both rodents^39^ and humans^53^ the occipital skull region, or more specifically the internal occipital protuberance, also contains a high density of channels that allow contact between the calvarial marrow and the CSF that is exiting the parenchyma and, during pathological states, enable immune messengers from the CNS to communicate with the periphery.^39^ The proximity of these channels to the confluence of sinuses may also contribute to the distinct patterns of immune activity observed in that region.

TSPO expression in the frontal skull was not found to be correlated with TSPO levels in the underlying tissues of the frontal cortex. This observation might seem unexpected considering prior indications of similar TSPO patterns in skull and adjacent brain regions in patients with Alzheimer’s disease.^36^ However, CSF serves as a dynamic communicator, playing a vital role not only in the immediate proximity but also in facilitating communication throughout the intricate network of the brain and its cranial environment. This finding also offers reassurance that the signals detected in the skull are not solely a consequence of partial volume effects of neighbouring tissues (a limitation associated with the PET scanner resolution).

Finally, we noted that depression group, as a statistical factor, was also a significant contributor to the increase in inflammation, specifically in the occipital skull and not in other extra-axial regions. This suggests that disease may modulate the local inflammatory response of the occipital skull through mechanisms beyond those considered in this study, involving factors beyond peripheral cytokines and neuroinflammation. Alternatively, the measurements of TNFα concentrations and parenchymal TSPO expression may not provide a comprehensive representation of peripheral or central immune activity associated with depression.

The mechanism that has been hypothesized here may be transdiagnostic. For example, occipital parameningeal tissue TSPO elevation has also been observed in patients with migraine with aura^40^ and may be the source of comorbidity across conditions (e.g. patients with depression have 2-fold chance of developing migraine,^56^ and there is a ±45% heritable association of migraine and major depressive disorder).^57^ Distinct patterns of TSPO signal elevation in the calvaria have also been observed in various pathologies, including multiple sclerosis, stroke, 4-repeat tauopathies and Alzheimer’s disease.^36^ In instances of central inflammation, a phenomenon observed across these pathologies, there is crosstalk between brain immune cells and immune cell reservoirs within the skull bone marrow, likely contributing to the elevated inflammatory signal that was previously observed in this depressed cohort.^9^

### Limitations

A few limitations should be considered in this study. Firstly, TSPO is expressed in numerous cell types, and there is considerable debate regarding the validity of its use as a conclusive marker for microglial activation in human cohorts. Other cell types that commonly express TSPO include astrocytes, activated macrophages, mast cells, neutrophiles, epithelial cells and vascular endothelial cells.^27-29^ However, it is important to note that these cell types are all implicated in central immune mechanisms. Therefore, although TSPO expression may not solely represent microglial activation, it is reasonable to conclude that elevations in TSPO expression observed in this study are conducive to inflammatory activity. Furthermore, given that this study examined the skull bone marrow, this inherently limits the possible TSPO-expressing cell types. Therefore, this strengthens our confidence that the TSPO signal expressed in the skull marrow is representative of immune activity.

Another possible limitation in this study lies in the use of the radiotracer ^11^C-PK11195. Although second-generation TSPO tracers, such as ^11^C-PBR28 and ^18^F-DPA-714, have been shown to have higher sensitivity to TSPO,^30^ they present challenges when used in humans. Approximately one-third of human subjects carry genetic polymorphisms within the *TSPO* gene, leading to low binding potential in second-generation tracers.^58^  ^11^C-PK11195 circumvents these challenges, enabling easier between-subject comparisons of TSPO binding. Importantly, numerous previous studies have validated the efficacy of ^11^C-PK11195 in measuring TSPO expression,^8^ supporting its utility in our investigation. The detection of significant group differences in our cohort using a less sensitive first-generation tracer adds robustness to our findings.

Furthermore, given that our ROIs are outside of the blood–brain barrier, we cannot be certain whether circulating radiometabolites are contributing to the measured signal. However, it is unlikely that radiometabolism varies significantly between groups. Although, to the best of our knowledge, differences in radiometabolism between depressed and healthy subjects have not been explored, this topic has been investigated in multiple sclerosis, where no significant differences in ^11^C-PK11195 plasma metabolism between groups were found.^59^ Given that these differences were not observed in an explicitly neuroinflammatory disorder, we have no reason to suspect that there would be differences in depressed versus healthy individuals. Additionally, we are not aware of ^11^C-PK11195 metabolites with affinity to TSPO, minimizing potential signal contamination even outside the brain.

## Conclusion

In conclusion, the findings of this study provide *in vivo* confirmation of observations from preclinical models, emphasizing the role of calvarial marrow in facilitating brain–periphery immune interactions within a human cohort. Furthermore, the occipital region might harbour a special immune cell niche that is reserved for supplying immune privilege to the brain during disease states, consistent with previous findings. These results also underscore the significance of the parietal and facial bone marrow and the venous sinuses as key locations for this crosstalk, thereby highlighting these regions as potential targets for future treatment strategies in central immune-related conditions.

## Supplementary Material

awae343_Supplementary_Data

## Data Availability

The data that support the findings of this study are available from the corresponding author upon reasonable request.

## References

[1] Miller AH, Raison CL. The role of inflammation in depression: From evolutionary imperative to modern treatment target. Nat Rev Immunol. 2016;16:22–34. 26711676 10.1038/nri.2015.5 PMC5542678

[2] Osimo EF, Pillinger T, Rodriguez IM, Khandaker GM, Pariante CM, Howes OD. Inflammatory markers in depression: A meta-analysis of mean differences and variability in 5,166 patients and 5,083 controls. Brain Behav Immun. 2020;87:901–909. 32113908 10.1016/j.bbi.2020.02.010 PMC7327519

[3] Dowlati Y, Herrmann N, Swardfager W, et al A meta-analysis of cytokines in major depression. Biol Psychiatry. 2010;67:446–457. 20015486 10.1016/j.biopsych.2009.09.033

[4] Goldsmith DR, Rapaport MH, Miller BJ. A meta-analysis of blood cytokine network alterations in psychiatric patients: Comparisons between schizophrenia, bipolar disorder and depression. Mol Psychiatry. 2016;21:1696–1709. 26903267 10.1038/mp.2016.3 PMC6056174

[5] Haapakoski R, Mathieu J, Ebmeier KP, Alenius H, Kivimaki M. Cumulative meta-analysis of interleukins 6 and 1β, tumour necrosis factor α and C-reactive protein in patients with major depressive disorder. Brain Behav Immun. 2015;49:206–215. 26065825 10.1016/j.bbi.2015.06.001 PMC4566946

[6] Köhler CA, Freitas TH, Maes M, et al Peripheral cytokine and chemokine alterations in depression: A meta-analysis of 82 studies. Acta Psychiatr Scand. 2017;135:373–387. 28122130 10.1111/acps.12698

[7] Setiawan E, Wilson AA, Mizrahi R, et al Role of translocator protein density, a marker of neuroinflammation, in the brain during major depressive episodes. JAMA Psychiatry. 2015;72:268–275. 25629589 10.1001/jamapsychiatry.2014.2427 PMC4836849

[8] Holmes SE, Hinz R, Conen S, et al Elevated translocator protein in anterior cingulate in major depression and a role for inflammation in suicidal thinking: A positron emission tomography study. Biol Psychiatry. 2018;83:61–69. 28939116 10.1016/j.biopsych.2017.08.005

[9] Schubert JJ, Veronese M, Fryer TD, et al A modest increase in ^11^C-PK11195-positron emission tomography TSPO binding in depression is not associated with serum C-reactive protein or body mass index. Biol Psychiatry Cogn Neurosci Neuroimaging. 2021;6:716–724. 33515765 10.1016/j.bpsc.2020.12.017 PMC8264953

[10] Li H, Sagar AP, Keri S. Translocator protein (18 kDa TSPO) binding, a marker of microglia, is reduced in major depression during cognitive-behavioral therapy. Prog Neuropsychopharmacol Biol Psychiatry. 2018;83:1–7. 29269262 10.1016/j.pnpbp.2017.12.011

[11] Setiawan E, Attwells S, Wilson AA, et al Association of translocator protein total distribution volume with duration of untreated major depressive disorder: A cross-sectional study. Lancet Psychiatry. 2018;5:339–347. 29496589 10.1016/S2215-0366(18)30048-8

[12] Richards EM, Zanotti-Fregonara P, Fujita M, et al PET radioligand binding to translocator protein (TSPO) is increased in unmedicated depressed subjects. EJNMMI Res. 2018;8:57. 29971587 10.1186/s13550-018-0401-9 PMC6029989

[13] Turkheimer FE, Veronese M, Mondelli V, Cash D, Pariante CM. Sickness behaviour and depression: An updated model of peripheral-central immunity interactions. Brain Behav Immun. 2023;111:202–210. 37076054 10.1016/j.bbi.2023.03.031

[14] Hodes GE, Ménard C, Russo SJ. Integrating interleukin-6 into depression diagnosis and treatment. Neurobiol Stress. 2016;4:15–22. 27981186 10.1016/j.ynstr.2016.03.003 PMC5146277

[15] Tanaka T, Narazaki M, Kishimoto T. IL-6 in inflammation, immunity, and disease. Cold Spring Harb Perspect Biol. 2014;6:a016295. 25190079 10.1101/cshperspect.a016295 PMC4176007

[16] Sforzini L, Cattaneo A, Ferrari C, et al Higher immune-related gene expression in major depression is independent of CRP levels: Results from the BIODEP study. Transl Psychiatry. 2023;13:185. 37264010 10.1038/s41398-023-02438-x PMC10235092

[17] Bauer ME, Teixeira AL. Inflammation in psychiatric disorders: What comes first? Ann N Y Acad Sci. 2019;1437:57–67. 29752710 10.1111/nyas.13712

[18] Raison CL, Demetrashvili M, Capuron L, Miller AH. Neuropsychiatric adverse effects of interferon-α. CNS Drugs. 2005;19:105–123. 15697325 10.2165/00023210-200519020-00002 PMC1255968

[19] Baraldi S, Hepgul N, Mondelli V, Pariante CM. Symptomatic treatment of interferon-α-induced depression in hepatitis C: A systematic review. J Clin Psychopharmacol. 2012;32:531–543. 22722514 10.1097/JCP.0b013e31825d9982

[20] Bai S, Guo W, Feng Y, et al Efficacy and safety of anti-inflammatory agents for the treatment of major depressive disorder: A systematic review and meta-analysis of randomised controlled trials. J Neurol Neurosurg Psychiatry. 2020;91:21–32. 31658959 10.1136/jnnp-2019-320912

[21] Raison CL, Rutherford RE, Woolwine BJ, et al A randomized controlled trial of the tumor necrosis factor antagonist infliximab for treatment-resistant depression: The role of baseline inflammatory biomarkers. JAMA Psychiatry. 2013;70:31–41. 22945416 10.1001/2013.jamapsychiatry.4 PMC4015348

[22] Nettis MA, Lombardo G, Hastings C, et al Augmentation therapy with minocycline in treatment-resistant depression patients with low-grade peripheral inflammation: Results from a double-blind randomised clinical trial. Neuropsychopharmacology. 2021;46:939–948. 33504955 10.1038/s41386-020-00948-6 PMC8096832

[23] Guzman-Martinez L, Maccioni RB, Andrade V, Navarrete LP, Pastor MG, Ramos-Escobar N. Neuroinflammation as a common feature of neurodegenerative disorders. Front Pharmacol. 2019;10:10. 31572186 10.3389/fphar.2019.01008 PMC6751310

[24] Liu GJ, Middleton RJ, Hatty CR, et al The 18 kDa translocator protein, microglia and neuroinflammation. Brain Pathol. 2014;24:631–653. 25345894 10.1111/bpa.12196 PMC8029074

[25] Banati RB, Newcombe J, Gunn RN, et al The peripheral benzodiazepine binding site in the brain in multiple sclerosis: Quantitative *in vivo* imaging of microglia as a measure of disease activity. Brain. 2000;123(Pt 11):2321–2337. 11050032 10.1093/brain/123.11.2321

[26] Nutma E, Fancy N, Weinert M, et al Translocator protein is a marker of activated microglia in rodent models but not human neurodegenerative diseases. Nat Commun. 2023;14:5247. 37640701 10.1038/s41467-023-40937-z PMC10462763

[27] Gui Y, Marks JD, Das S, Hyman BT, Serrano-Pozo A. Characterization of the 18 kDa translocator protein (TSPO) expression in post-mortem normal and Alzheimer’s disease brains. Brain Pathol. 2020;30:151–164. 31276244 10.1111/bpa.12763 PMC6904423

[28] Rupprecht R, Papadopoulos V, Rammes G, et al Translocator protein (18 kDa) (TSPO) as a therapeutic target for neurological and psychiatric disorders. Nat Rev Drug Discov. 2010;9:971–988. 21119734 10.1038/nrd3295

[29] Owen DR, Narayan N, Wells L, et al Pro-inflammatory activation of primary microglia and macrophages increases 18 kDa translocator protein expression in rodents but not humans. J Cereb Blood Flow Metab. 2017;37:2679–2690. 28530125 10.1177/0271678X17710182 PMC5536262

[30] Vicente-Rodriguez M, Singh N, Turkheimer F, et al Resolving the cellular specificity of TSPO imaging in a rat model of peripherally-induced neuroinflammation. Brain Behav Immun. 2021;96:154–167. 34052363 10.1016/j.bbi.2021.05.025 PMC8323128

[31] Turkheimer FE, Althubaity N, Schubert J, et al Increased serum peripheral C-reactive protein is associated with reduced brain barriers permeability of TSPO radioligands in healthy volunteers and depressed patients: Implications for inflammation and depression. Brain Behav Immun. 2021;91:487–497. 33160089 10.1016/j.bbi.2020.10.025

[32] Althubaity N, Schubert J, Martins D, et al Choroid plexus enlargement is associated with neuroinflammation and reduction of blood brain barrier permeability in depression. Neuroimage Clin. 2022;33:102926. 34972034 10.1016/j.nicl.2021.102926 PMC8718974

[33] Cai R, Pan C, Ghasemigharagoz A, et al Panoptic imaging of transparent mice reveals whole-body neuronal projections and skull-meninges connections. Nat Neurosci. 2019;22:317–327. 30598527 10.1038/s41593-018-0301-3 PMC6494982

[34] Cugurra A, Mamuladze T, Rustenhoven J, et al Skull and vertebral bone marrow are myeloid cell reservoirs for the meninges and CNS parenchyma. Science. 2021;373:eabf7844. 34083447 10.1126/science.abf7844 PMC8863069

[35] Herisson F, Frodermann V, Courties G, et al Direct vascular channels connect skull bone marrow and the brain surface enabling myeloid cell migration. Nat Neurosci. 2018;21:1209–1217. 30150661 10.1038/s41593-018-0213-2 PMC6148759

[36] Kolabas ZI, Kuemmerle LB, Perneczky R, et al Distinct molecular profiles of skull bone marrow in health and neurological disorders. Cell. 2023;186:3706–25.e29. 37562402 10.1016/j.cell.2023.07.009 PMC10443631

[37] Lynall M-E, Kigar SL, Lehmann ML, et al B-cells are abnormal in psychosocial stress and regulate meningeal myeloid cell activation. Brain Behav Immun. 2021;97:226–238. 34371135 10.1016/j.bbi.2021.08.002 PMC8453122

[38] Brioschi S, Wang WL, Peng V, et al Heterogeneity of meningeal B cells reveals a lymphopoietic niche at the CNS borders. Science. 2021;373:eabf9277. 34083450 10.1126/science.abf9277 PMC8448524

[39] Pulous FE, Cruz-Hernandez JC, Yang C, et al Cerebrospinal fluid can exit into the skull bone marrow and instruct cranial hematopoiesis in mice with bacterial meningitis. Nat Neurosci. 2022;25:567–576. 35501382 10.1038/s41593-022-01060-2 PMC9081225

[40] Hadjikhani N, Albrecht DS, Mainero C, et al Extra-axial inflammatory signal in parameninges in migraine with visual aura. Ann Neurol. 2020;87:939–949. 32239542 10.1002/ana.25731 PMC7313427

[41] Chamberlain SR, Cavanagh J, de Boer P, et al Treatment-resistant depression and peripheral C-reactive protein. Br J Psychiatry. 2019;214:11–19. 29764522 10.1192/bjp.2018.66 PMC6124647

[42] Prados F, Cardoso MJ, Burgos N, Gandini Wheeler-Kingshott CAM, Ourselin S. NiftyWeb: Web based platform for image processing on the cloud. In: *24th Scientific Meeting and Exhibition of the International Society for Magnetic Resonance in Medicine*. ISMRM. 2016:abstract 1049.

[43] Burgos N, Cardoso MJ, Thielemans K, et al Attenuation correction synthesis for hybrid PET-MR scanners: Application to brain studies. IEEE Trans Med Imaging. 2014;33:2332–2341. 25055381 10.1109/TMI.2014.2340135

[44] Manavaki R, Hong YT, Fryer TD. Brain MRI coil attenuation map processing for the GE SIGNA PET/MR: Impact on PET image quantification and uniformity. In: *2019 IEEE Nuclear Science Symposium and Medical Imaging Conference (NSS/MIC)*. IEEE. 2019.

[45] Maccioni L, Michelle CM, Brusaferri L, et al A blood-free modeling approach for the quantification of the blood-to-brain tracer exchange in TSPO PET imaging. Front Neurosci. 2024;18:1395769. 39104610 10.3389/fnins.2024.1395769 PMC11299498

[46] Browning H . The confluence of dural venous sinuses. Am J Anat. 1953;93:307–329. 13104335 10.1002/aja.1000930302

[47] Eisová S, Píšová H, Velemínský P, Bruner E. Normal craniovascular variation in two modern European adult populations. J Anat. 2019;235:765–782. 31236921 10.1111/joa.13019 PMC6742892

[48] Patel N . Venous anatomy and imaging of the first centimeter. Semin Ultrasound CT MR. 2009;30:513–524. 20099637 10.1053/j.sult.2009.08.003

[49] Rustenhoven J, Drieu A, Mamuladze T, et al Functional characterization of the dural sinuses as a neuroimmune interface. Cell. 2021;184:1000–16.e27. 33508229 10.1016/j.cell.2020.12.040 PMC8487654

[50] Aspelund A, Antila S, Proulx ST, et al A dural lymphatic vascular system that drains brain interstitial fluid and macromolecules. J Exp Med. 2015;212:991–999. 26077718 10.1084/jem.20142290 PMC4493418

[51] Sandiego CM, Gallezot JD, Pittman B, et al Imaging robust microglial activation after lipopolysaccharide administration in humans with PET. Proc Natl Acad Sci U S A. 2015;112:12468–12473. 26385967 10.1073/pnas.1511003112 PMC4603509

[52] Bruner E, Eisová S. Vascular microforamina and endocranial surface: Normal variation and distribution in adult humans. Anat Rec. 2024;307:3375–3383 10.1002/ar.25426 38465854

[53] Rangel-de Lázaro G, Neubauer S, Gunz P, Bruner E. Ontogenetic changes of diploic channels in modern humans. Am J Phys Anthropol. 2020;173:96–111. 32462711 10.1002/ajpa.24085

[54] Thangaleela S, Sivamaruthi BS, Kesika P, Bharathi M, Chaiyasut C. Nasal microbiota, olfactory health, neurological disorders and aging—A review. Microorganisms. 2022;10:1405. 35889124 10.3390/microorganisms10071405 PMC9320618

[55] Kumpitsch C, Koskinen K, Schopf V, Moissl-Eichinger C. The microbiome of the upper respiratory tract in health and disease. BMC Biol. 2019;17:87. 31699101 10.1186/s12915-019-0703-z PMC6836414

[56] Castelnuovo G, Giusti EM, Manzoni GM, et al Psychological considerations in the assessment and treatment of pain in neurorehabilitation and psychological factors predictive of therapeutic response: Evidence and recommendations from the Italian consensus conference on pain in neurorehabilitation. Front Psychol. 2016;7:468. 27148104 10.3389/fpsyg.2016.00468 PMC4835496

[57] May A, Schulte LH. Chronic migraine: Risk factors, mechanisms and treatment. Nat Rev Neurol. 2016;12:455–464. 27389092 10.1038/nrneurol.2016.93

[58] Owen DR, Yeo AJ, Gunn RN, et al An 18-kDa translocator protein (TSPO) polymorphism explains differences in binding affinity of the PET radioligand PBR28. J Cereb Blood Flow Metab. 2012;32:1–5. 22008728 10.1038/jcbfm.2011.147 PMC3323305

[59] de Souza AM, Pitombeira MS, de Souza LE, et al ^11^C-PK11195 plasma metabolization has the same rate in multiple sclerosis patients and healthy controls: A cross-sectional study. Neural Regen Res. 2021;16:2494–2498. 33907039 10.4103/1673-5374.313062 PMC8374550

