# Supplementary material for: Extra-axial inflammatory signal and its relationship to peripheral and central immunity in depression
Source: Brain. 2024 Dec 10;148(2):635–46. doi: 10.1093/brain/awae343 (PMC11788198; doi:10.1093/brain/awae343)
Supplement: awae343_Supplementary_Data [file awae343_supplementary_data.zip › brain-2024-00496-file010.pdf]

Extra-axial inflammatory signal and its relation to peripheral and central immunity in depression

Brandi Eiff et al.

Supplemental material

Supplemental figures

Supplementary Fig. 1 is supplied as a separate file.

Supplementary Fig. 1. 3D Representation of Skull Regions of Interest. Three-dimensional rendering depicting specific regions of interest within the skull. Regions of interest were informed by the template provided by Hadjikhani et al[40] and pseudo CT renderings derived from participants' structural MRIs. The use of pseudo CTs facilitated the refinement of these regions, ensuring that voxels exclusively represented bone marrow without including adjacent dura mater. DLPFC = dorsolateral prefrontal cortex, MRI = magnetic resonance imaging.

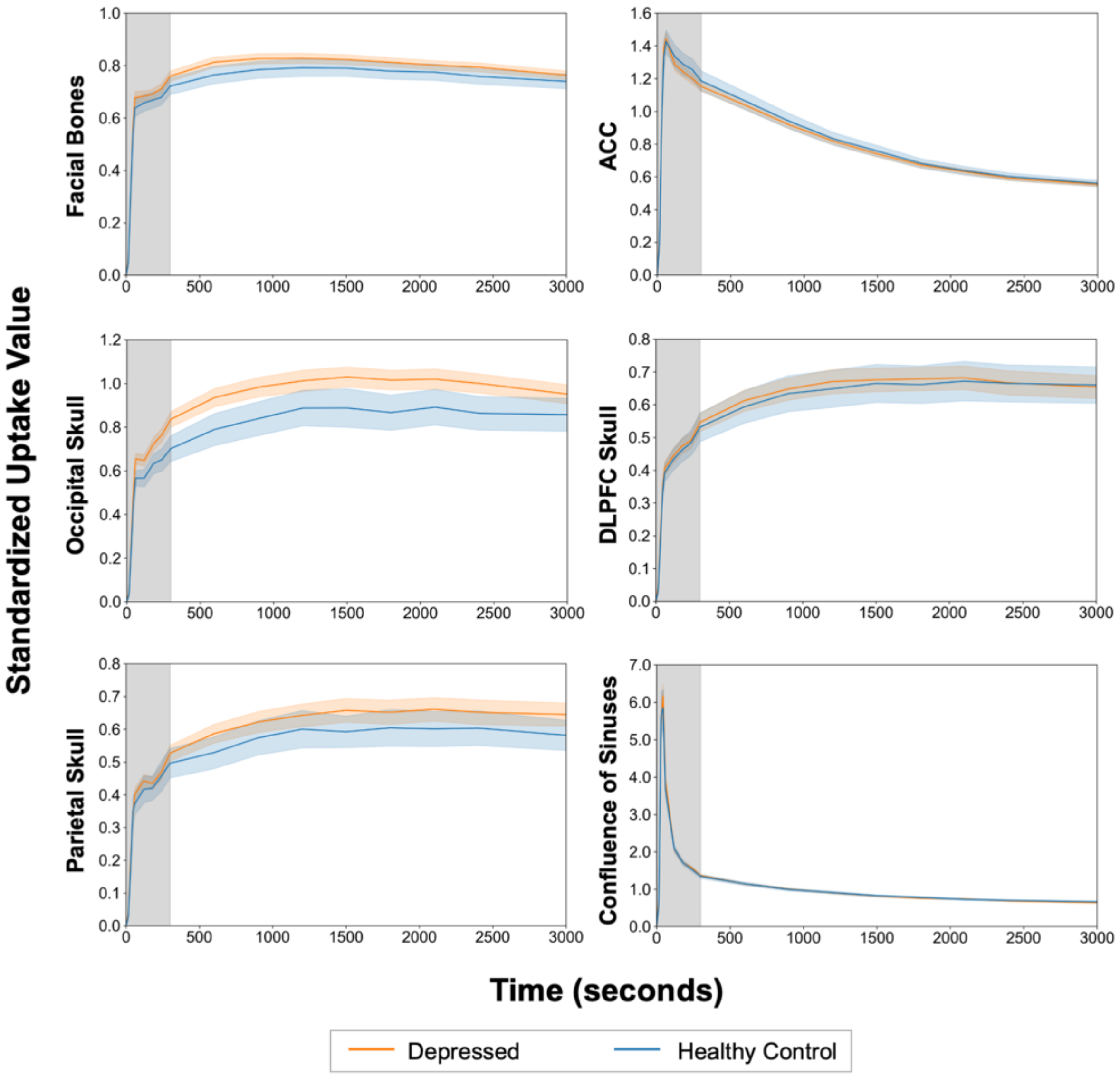

Supplementary Fig. 2. Average Standardized Uptake Value (SUV) Curves for Central and Extra-Axial Regions of Interest. The average SUV curves for healthy controls (blue) and depressed subjects (orange) are presented across the regions of interest: facial bones, occipital skull, parietal skull, anterior cingulate cortex (ACC), dorsolateral prefrontal cortex (DLPFC), and the confluence of sinuses. Standard error is depicted as the shaded area around each group's curves. The grey-shaded area represents the time period excluded from the average SUV calculations.

Supplemental methods

A binary mask for the carotid siphons was created by identifying voxels with PET kinetic activity that resembled blood within the middle cranial fossa. This process involved intensity thresholding of the early dynamic PET frames. For each subject, time-activity curves (TACs) were extracted from the carotid siphon region, and ^{11}C-PK11195 binding was quantified using standardized uptake values (SUVs). The TAC data used for quantification ranged from 5 minutes after the start of the scan until the end of the scan period.

A repeated measures general linear model (GLM) was employed, treating extra-axial regional average SUV values (facial bones, occipital skull, DLPFC skull, parietal skull, and carotid siphons) as the repeated measure, subject group as the fixed factor, and logTNF-α and ACC SUV as covariates. A univariate analysis of covariance (ANCOVA) was conducted for the average carotid siphons SUV, with subject's group as the fixed factor and logTNF-α and ACC SUV as covariates.

Supplemental results

Repeated measures GLM

Mauchly's test of sphericity indicated that the assumption of sphericity was violated [$\chi^2(9) = 71.206$, p <0.001]; therefore, Greenhouse-Geisser correction was applied ($\varepsilon = 0.673$) to the model. TNF-α, ACC SUV, and group significantly contributed to variation in extra-axial inflammation between subjects [TNF-α: $F(1, 67) = 4.118$, $p = 0.046$, $\eta p^2 = 0.058$; ACC SUV: $F(1, 67) = 10.454$, $p = 0.002$, $\eta p^2 = 0.135$; group: $F(1, 67) = 4.648$, $p = 0.035$, $\eta p^2 = 0.065$]. No interaction effects were observed between the extra-axial regions and other variables when the carotid siphons were included in the model in place of the confluence of sinuses.

Univariate ANCOVA

ACC SUV had a significant positive effect on SUV within the carotid siphons [$F(1,67) = 12.521$, $p < 0.001$, $\eta p^2 = 0.157$] while, unlike within the confluence of sinuses, TNF-α did not have a significant effect on SUV variance within the carotid siphons [$F(1,67) = 1.843$, $p = 0.179$, $\eta p^2 = 0.027$].
